# Dietary patterns among U.S. food insecure cancer survivors and the risk of mortality: NHANES 1999–2018

**DOI:** 10.1007/s10552-024-01868-2

**Published:** 2024-03-26

**Authors:** Christian A. Maino Vieytes, Ruoqing Zhu, Francesca Gany, Brenda D. Koester, Anna E. Arthur

**Affiliations:** 1 Division of Nutritional Sciences, University of Illinois at Urbana-Champaign, 386 Bevier Hall, 905 S Goodwin Ave, Urbana, IL 61801 USA; 2 Department of Statistics, University of Illinois at Urbana-Champaign, Urbana, IL 61801 USA; 3 Memorial Sloan Kettering Cancer Center, New York, NY 10065 USA; 4 Family Resiliency Center, University of Illinois at Urbana-Champaign, Urbana, IL 61801 USA; 5 Department of Dietetics and Nutrition, Medical Center, University of Kansas, Kansas City, KS 66160 USA

**Keywords:** Nutritional epidemiology, Survivorship, Dietary patterns, Food insecurity, Regularization

## Abstract

**Purpose:**

Food insecurity—the lack of unabated access to nutritious foods—is a consequence many cancer survivors face. Food insecurity is associated with adverse health outcomes and lower diet quality in the general public. The goal of this analysis was to extract major and prevailing dietary patterns among food insecure cancer survivors from observed 24-h recall data and evaluate their relationship to survival after a cancer diagnosis.

**Methods:**

We implemented two dietary patterns analysis approaches: penalized logistic regression and principal components analysis. Using nationally representative data from the National Health and Nutrition Examination Survey (NHANES) study, we extracted three dietary patterns. Additionally, we evaluated the HEI-2015 for comparison. Cox proportional hazards models assessed the relationship between the diet quality indices and survival after a cancer diagnosis.

**Results:**

There were 981 deaths from all causes and 343 cancer-related deaths. After multivariable adjustment, we found higher risks of all-cause mortality associated with higher adherence to Pattern #1 (HR 1.25; 95% CI 1.09–1.43) and Pattern #2 (HR 1.15; 95% CI 1.01–1.31) among cancer survivors.

**Conclusion:**

Among all cancer survivors, higher adherence to major and prevailing dietary patterns from the U.S. food insecure cancer survivor population may lead to worse survival outcomes.

**Supplementary Information:**

The online version contains supplementary material available at 10.1007/s10552-024-01868-2.

## Introduction

A cancer diagnosis can upend several facets of life and well-being. In addition to psychological distress from the diagnosis, financial toxicity and its accompanying distress can emerge for many cancer survivors owing to exorbitant treatment, prescription, and indirect costs (e.g., income loss due to cancer-related job loss or disability) [1]. These phenomena we describe are often magnified for cancer survivors—defined as individuals with a history of cancer-with low income who may lack financial reserves and workplace accommodations while navigating the treatment phases of their cancer [1, 2].

Food insecurity, or the lack of continuous access to healthy and nutritious foods to lead a healthy life, can be a consequence for cancer survivors facing high financial toxicity burdens [3, 4]. A framework of competing demands conceptualizes one manifestation of food insecurity among cancer survivors, involving cancer survivors facing difficult decisions between choosing medical care or nutritious foods [3]. A critical public health concern is that food insecurity is associated with adverse health outcomes and lower dietary quality in the general public [5, 6]. Food insecurity may predict a worse prognosis among cancer survivors, though the evidence is limited, and more research is needed to substantiate this conjecture.

Seligman and Schillinger proposed a conceptual framework for how food insecurity may relate to adverse health outcomes with diet quality as a mediating variable [5]. Understanding how food insecurity affects different aspects of life, including dietary intake behaviors, is a means of delineating at least one potential driving factor behind the health disparities that may arise in cancer survivors experiencing food insecurity. Therefore, the goal of this analysis was to use nationally representative data to examine associations between dietary patterns in the food insecure cancer survivor population and the risk of mortality. We used several tools for characterizing dietary patterns from observed 24-h recall data to understand the major dietary patterns among food insecure cancer survivors. We hypothesized that these dietary patterns describing consumption patterns in the food insecure cancer survivor population would be positively associated with mortality in cancer survivors and food insecure cancer survivors.

## Methods

### Study setting and population

We employed data from ten consecutive cycles (1999–2018) of the NHANES, a biennial cross-sectional study implemented by the Centers for Disease Control and Prevention (CDC) and the National Center for Health Statistics (NCHS), which sampled civilian and non-institutionalized community dwellers in the United States. The study implements a complex multi-stage sampling design that generates a nationally representative sample and aims to characterize the relationships between lifestyle, medical, environmental, and other factors and health outcomes. It uses surveys that span numerous facets of health and lifestyle and includes a medical examination for a subset of participants.

**Fig. 1:**
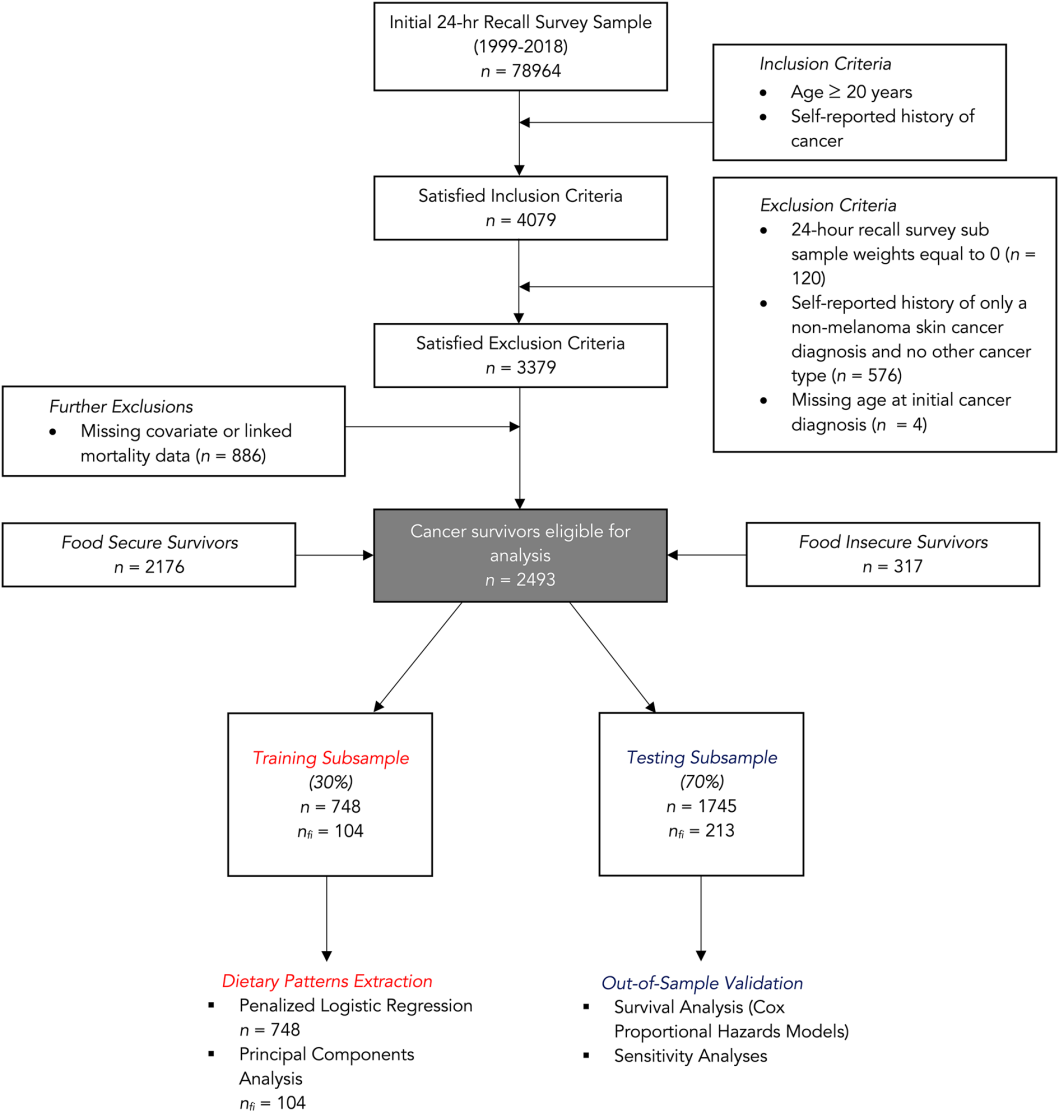
Sample flow diagram detailing inclusion and exclusion criteria for arriving at the final sample and the overall analytical strategy. *n*_*fi*_* = food insecure subset sample size.*

In Fig. 1, we detail the sample flow that arrived at the final analytical sample of cancer survivors (*n* = 2,493), which included food secure participants (*n* = 2,176) and food insecure participants (*n* = 317). We divided the final sample into randomly generated subsets that would be used for training machine learning models and the validation analysis. We discuss this division with greater detail in the coming sections. Food insecurity status was measured using the U.S. Department of Agriculture (USDA) U.S. Food Security Survey Module (U.S. FSSM), which consists of 18 items designed to evaluate the degree of food insecurity experienced by a participant’s household over the preceding year [7]. The survey consists of a series of “yes/no” questions, and responses in the affirmative are used to categorize a household as food insecure (responding in the affirmative to ≥ three items) or food secure (responding in the affirmative to ≤ two items). Cancer history was ascertained via self-reporting using the Medical Conditions Questionnaire (MCQ). Individuals with a history of non-melanoma skin cancer and no other cancer were coded as having no history of cancer, given that the prognosis and benign course of this class of malignancies may otherwise bias the sample [8].

### Explanatory variables: diet quality measures

NHANES study staff assessed dietary intake through two 24-h recalls using the USDA Automated Multiple-Pass Method [9, 10]. For cycles between 1999 and 2002, only a single 24-h recall was performed. Nutrient intake data were estimated by referencing the Food and Nutrient Database for Dietary Studies (FNDDS). Dietary and nutrient intake data were averaged across both 24-h recalls as previously described [11–13]. We used the USDA Food Patterns Equivalents Database (FPED) and the MyPyramid Equivalents Database (MPED) to obtain intake equivalents of 37 USDA food pattern components, collapsed further into 26 groups, as previously described [11]. Empirical diet quality measures were extracted from observed dietary data using penalized logistic regression (penalized logit) and principal components analysis (PCA). The 26 food groups were the explanatory variables in these models (see Table 2 for the food groups used in this analysis). In the case of the penalized logit model, food insecurity status (*food insecure*/*food secure*) was regressed on the centered and scaled transformations of the food group variables and total calories—i.e., the latter was included to implement the standard multivariate method for total energy adjustment described by Willett et al. [14]. We trained this model on a random sample (referred to as the “training subsample” henceforth) of *n* = 748 subjects (30% of the original sample—see Fig. 1). The dietary pattern extracted using this method was assigned *Pattern #1*. PCA for dietary patterns extraction was performed on the food insecure subjects of this training subsample (*n* = 104). We used a scree plot to determine the number of components to retain. Two components were retained and assigned *Patterns #2* and *#3*. The out-of-sample validation analysis (see *Statistical Analysis* section below) was performed on the remaining fraction (*n* = 1,745—comprising 70% of the original sample) of subjects (referred to as the “testing subsample” henceforth). The supplementary methods file contains additional details about these procedures. For the sake of comparison, we also computed Healthy Eating Index 2015 (HEI-2015) scores and incorporated them into all subsequent analyses [15, 16]. The dietary patterns scores generated with PCA and the HEI-2015 scores were energy-adjusted using the residual method [14].

### Response variables: all-cause and cause-specific mortalities

Mortality and time-to-event data were acquired from the NHANES Public-Use Linked Mortality File, which was generated from deterministic and probabilistic linkages of the NHANES survey data (through the 2017–2018 cycle) to the National Death Index, as described elsewhere [17]. We computed time since diagnosis, defined as the difference between the age at the time of the survey and the age at the first cancer diagnosis, and used it as the time scale in our models to minimize potential bias by accounting for left truncation due to delayed study enrollment following diagnosis [18, 19]. Data were right-censored to either the last known date alive or the administrative censoring date on December 31, 2019. We used the International Classification of Diseases, Tenth Revision (ICD-10) codes to classify the causes of death. Survival analyses examined all-cause mortality and cause-specific mortality—deaths due to neoplastic malignancy (ICD-10 codes C00-C97).

### Covariates

Self-reported demographic and socioeconomic data were obtained during the home interview. Characteristics from the demographic survey (DEMO) included age, sex (*male*/*female*), race and ethnicity (*Mexican-American*, *Other Hispanic*, *non-Hispanic White, non-Hispanic Black,* and *Other/Multiracial*), family income-to-poverty ratio (< 1.3 or ≥ 1.3), and household size. We obtained health insurance status data (*covered by health insurance*/*not covered by health insurance*) from the Health Insurance Questionnaire (HIQ/HID). Behavioral characteristics included smoking status (*current smoker*—currently smoking every day or some days—, *former smoker*—not currently smoking but with a lifetime history of ≥ 100 cigarettes—, or *never smoker*—a lifetime history of smoking < 100 cigarettes), drinking status (*heavy drinker*— ≥ 14 g/day for women and ≥ 28 g/day for men—, *moderate drinker*—0.10–13.9 g/day for women and 0.10–27.9 g/day for men—, and *abstainer*— < 0.10 g/day), and physical activity (*weekly MET minutes*). These data were obtained from the Smoking Questionnaire (SMQ), 24-h recalls, and physical activity questionnaires (PAQ and PAQIAF), respectively. Health-related covariates included the Charlson Comorbidity Index score (adapted for NHANES) and body mass index (BMI—kg/m^2^) measured during the physical examination [20]. Physical disability was assessed using the 19-item and validated NHANES Activities of Daily Living (ADL) scale from the Physical Functioning Questionnaire (PFQ), which is described in detail elsewhere [21]. Cancer-related covariates were obtained from the MCQ.

### Statistical analysis

We assessed the relationship between diet quality measures and all-cause and cause-specific mortality in an out-of-sample validation analysis using Cox Proportional Hazards models performed on the *n* = 1,745 subjects not used to extract the dietary patterns (the testing subsample—Fig. 1). We implemented several model specifications for the conditional log hazard function to assess the robustness of our results (Eqs. 1–4).

1 $$\lambda \left( {t {|} z_{ki} , v_{i} } \right) = \lambda_{0} \left( t \right)\exp \left( {\mathop \sum \limits_{k = 1}^{K - 1} \beta_{k} z_{ki} + \gamma v_{i} } \right)$$

2 $$\begin{gathered} \lambda \left( {t | a_{i} , v_{i} } \right) = \lambda_{0} \left( t \right)\exp \left( {\beta a_{i} + \gamma v_{i} } \right), \hfill \\ {\text{where}}\, a_{i} = \mathop \sum \limits_{k = 1}^{K} z_{ki} *{\text{median}}\left( {x_{k} } \right),\,{\text{and}}\,x_{k} \subseteq x \hfill \\ \end{gathered}$$

3 $$\begin{gathered} \lambda \left( {t | x_{i} ,v_{i} } \right) = \lambda_{0} \left( t \right)\exp \left( {\beta \frac{{x_{i} }}{\delta } + \gamma v_{i} } \right), \hfill \\ {\text{where}}\,\delta = {\text{SD}}\left( x \right) \hfill \\ \end{gathered}$$

4 $$\lambda \left( {t | x_{i} ,v_{i} } \right) = \lambda_{0} \left( t \right)\exp \left( {\mathop \sum \limits_{m = 1}^{M} \beta_{m} h_{m} \left( {x_{i} } \right) + \gamma v_{i} } \right)$$

The model in Eq. 1 specifies the diet quality index using *K* − 1 dummy variables, ${z}_{ki}$, which indicates the *i*th subject’s membership in one of the quintiles ($K=5$) of the dietary pattern index score. In Eq. 2, we conduct a trend test by assigning the *i*th subject the median of their respective quintile (where ${x}_{k}$ is the set of diet quality index scores for subjects in the *k*th quintile) and then modeling it as a continuous variable (${a}_{i}$). In Eq. 3, we specify the diet index as a continuous variable scaled by the standard deviation of the index and in Eq. 3 we specify the diet index (${x}_{i}$) with a basis expansion of $M=3$ basis functions (see the supplementary methods file) for a natural cubic spline. The model fit using Eq. 4 used one interior knot (${\xi }_{{\ell}}$). Given that Model 3 is nested in Model 4, we used the likelihood ratio test to assess for non-linearity [22]. Additionally, all models included parameters ($\gamma$) for covariates ($v$). We fit these models to data from the entire sample of cancer survivors in the testing subsample (*n* = 1,745) and on food insecure cancer survivors only (*n* = 213). Furthermore, we used sequential adjustment within these model specifications. A *Null* model did not adjust for any covariates beyond the dietary pattern index score. A *Basic* model further adjusted for age, sex, and race and ethnicity. A *Full* model further adjusted for BMI, household size, family income-to-poverty ratio, education status, health insurance status, alcohol intake, smoking status, calories, weekly MET minutes, Charlson Comorbidity Index score, food insecurity status, and receipt of SNAP benefits [14, 23]. Covariates were selected a priori based on previous literature and working knowledge of potential confounders in the hypothesized pathway. To account for the possibility of downwardly biased survival estimates from the contributions of participants distantly removed from a cancer diagnosis to the risk set, we conducted a sensitivity analysis including only participants with a primary cancer diagnosis within the four years preceding their interview date (*n* = 535). We also considered the NHANES ADL score as a covariate, given that food insecurity can be associated with physical disability and functional deficits. However, given the significant missingness in this variable, we did not include it in our primary models. Instead, we conducted a sub-analysis where we further adjusted for the NHANES ADL score. Finally, as an additional robustness test, we conducted propensity score matching to refine the sample to sets of new samples where covariate distributions were similar across high and low scores of the dietary patterns (see the supplementary methods files for more details). We then refit the survival models on those samples. All analyses accounted for the complex and probability-based sampling methods of the NHANES study by following the analytical guidelines provided by the NCHS and weighting them accordingly. We used $\alpha$ = 0.05 as our threshold level for statistical significance and performed all analyses in R v4.2.2 (The R Foundation, Vienna, Austria). The R code and data to reproduce these analyses are publicly accessible at: https://github.com/cmainov/nhanes-fi-ca-mortality (Accessed 21 March 2024).

## Results

The analysis included 603,960 person-months of contribution to the risk set, with 981 deaths from all causes, 343 cancer deaths, and 235 cardiovascular disease-related deaths. The characteristics of the study sample of cancer survivors stratified on food insecurity status are presented in Table 1. On average, food insecure cancer survivors in this sample were younger than food secure survivors, more likely to be female, Non-Hispanic Black, Hispanic, multiracial, have a lower educational status, live under the poverty line, and less likely to be covered by health insurance. Food insecure cancer survivors were also more likely to live in a home with five or more individuals, have a physical or functional impairment, identify as current smokers, have a greater comorbidity burden, and were less likely to be heavy drinkers than their food secure counterparts.

**Table 1:** Epidemiologic characteristics of the study sample

Characteristic	Combined sample (*n* = 2,493)	Food insecure (*n* = 317)	Food secure (*n* = 2176)	***p***
Age—mean (SD)	62.03 (14.85)	50.4 (16.46)	63.32 (14.09)	< 0.01
Sex				< 0.01
Male	1,139 (40.9)	99 (25.1)	1040 (42.6)	
Female	1,354 (59.1)	218 (74.9)	1136 (57.4)	
Race/ethnicity				< 0.01
Mexican-American	174 (2.3)	51 (8.0)	123 (1.7)	
Other Hispanic	133 (2.5)	40 (7.5)	93 (1.9)	
Non-Hispanic White	1,730 (86.5)	156 (70.6)	1574 (88.3)	
Non-Hispanic Black	376 (6.2)	56 (9.2)	320 (5.9)	
Other/multiracial	80 (2.4)	14 (4.6)	66 (2.1)	
Education attained				< 0.01
≤ High school	1,197 (36.5)	205 (55.5)	992 (34.4)	
≥ Some college	1,296 (63.5)	112 (44.5)	1184 (65.6)	
Family income to poverty ratio				< 0.01
< 1.3	637 (17.3)	221 (63.5)	416 (12.2)	
Health insurance status				< 0.01
Insured	2,329 (94.1)	265 (83.7)	2064 (95.3)	
Household Size				< 0.01
< 5 Persons	2,274 (92.5)	247 (78.6)	2027 (94.1)	
≥ 5 Persons	219 (7.5)	70 (21.4)	149 (5.9)	
NHANES ADL Score—Mean (SD)	22.26 (4.56)	26.1 (7.34)	21.93 (4.07)	< 0.01
BMI (kg/m^2^)—Mean (SD)	28.92 (6.61)	29.82 (7.43)	28.82 (6.51)	0.08
Weekly MET Minutes—Mean (SD)	2,249.04 (4,387.81)	5,,195.27 (8691.45)	1923.51 (3462.9)	< 0.01
Daily Caloric Intake (kcal)—Mean (SD)	1,900.17 (679.88)	1,751 (791.25)	1916.65 (664.54)	0.02
Charlson Comorbidity Index—Mean (SD)	2.98 (1.35)	3.36 (1.71)	2.94 (1.3)	< 0.01
SNAP benefits				< 0.01
Receiving	347 (11.2)	158 (55.6)	189 (6.3)	
Years since diagnosis				0.84
≤ 4 years	753 (26.6)	104 (25.9)	649 (26.7)	
> 4 years	1,740 (73.4)	213 (74.1)	1527 (73.3)	
Smoking status				< 0.01
Current	393 (16.9)	107 (39.2)	286 (14.4)	
Former	1,021 (39.4)	79 (21.9)	942 (41.3)	
Never	1,079 (43.7)	131 (38.9)	948 (44.2)	
Alcohol use				0.05
Heavy	268 (13.8)	23 (4.6)	245 (14.9)	
Moderate	381 (15.9)	32 (16.1)	349 (15.9)	
None	1,844 (70.3)	262 (79.3)	1582 (69.3)	
Cause of death				0.42
Cancer	343 (36.4)	30 (31.9)	313 (36.7)	
Cardiovascular Dis.	235 (25.6)	11 (20.4)	224 (25.9)	
Other	403 (38.0)	41 (47.7)	362 (37.4)	

Percentages may not add to 100% given rounding

Raw sample frequencies are presented for categorical variables, but percentages (in parentheses) are survey-weighted estimates; i.e., they reflect population (and not sample) percentages

*p-*values are from ${\chi }^{2}$ tests for categorical variables and *t*-tests for continuous variables

Subjects were weighted, and the analysis was performed according to NCHS guidelines

**Fig. 2:**
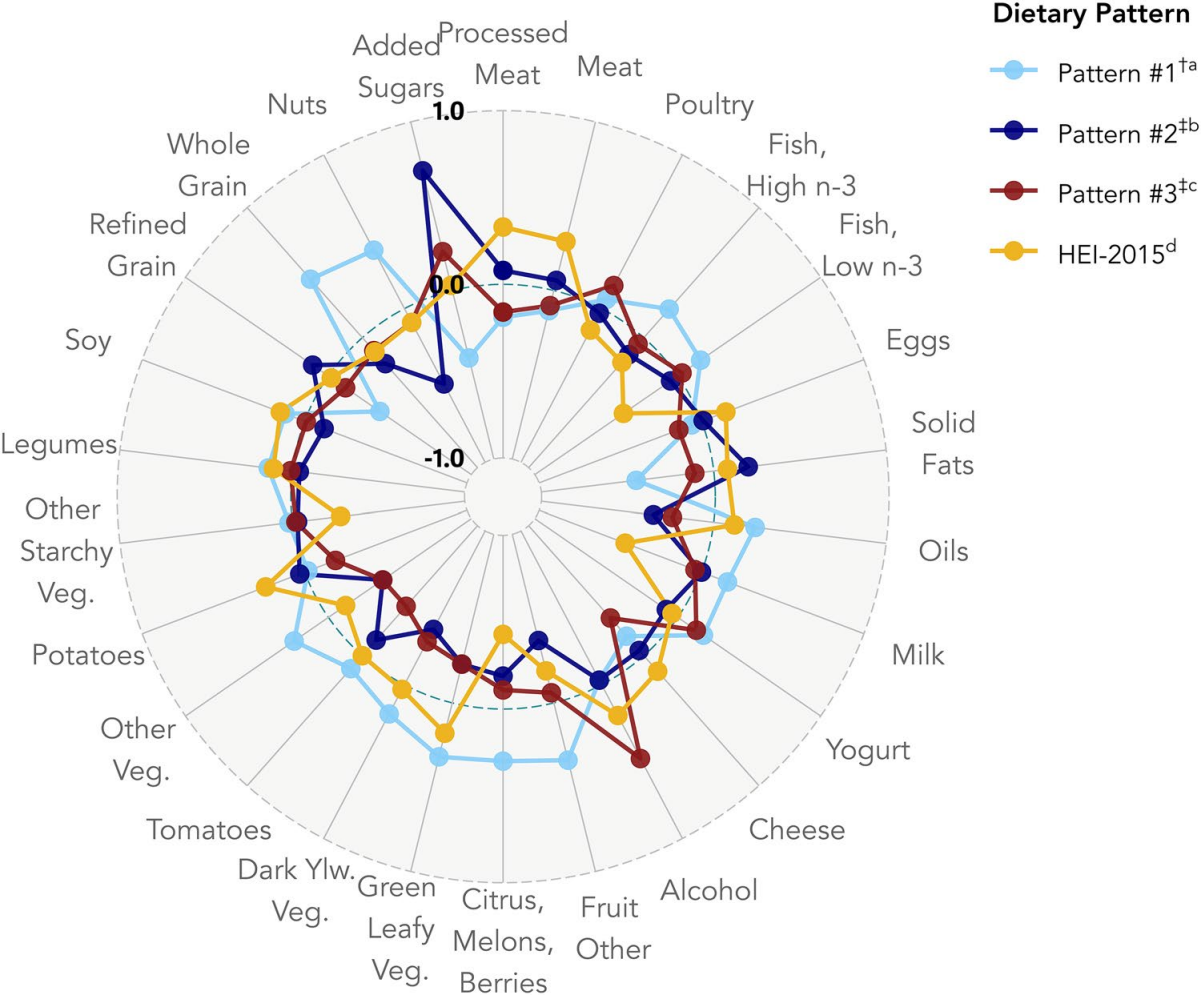
Radar chart with overlay of select dietary patterns and their correlations to the 26 food groups used in the dietary patterns extraction phase of the analysis. ^a^The High Fat & Sugar, Low Vegetable Pattern. ^b^The High Alcohol & Added Sugar Pattern. ^c^The Meat & Potatoes Pattern. ^d^Healthy Eating Index 2015. ^†^Dietary pattern obtained using penalized logistic regression; ^‡^Dietary pattern obtained using principal components analysis. Subjects were weighted, and the analysis was performed according to NCHS guidelines. The correlation analysis was performed on subjects from the testing subsample described in the main text (*n* = 1,745). All dietary patterns extraction procedures were performed on the training subsample described in the main text (*n* = 748).

Table 2 and Fig. 2 present weighted Pearson correlation coefficients between the extracted dietary patterns and the individual food groups comprising them. Pattern #1 (generated using penalized logit) was characterized by negative correlations with fruits, vegetables, oils, nuts, and whole grains, a high correlation with added sugars, and a weak-to-moderate positive correlation with solid fats. We otherwise refer to this pattern as the “High Fat & Sugar, Low Vegetable” pattern. We retained two dietary patterns from the PCA. Pattern #2 was negatively correlated with the consumption of oils, cheese, vegetables, and tomatoes and positively correlated with added sugar consumption and alcohol intake. Pattern #3 was positively correlated with the consumption of processed meats and other meats, alcohol, and potatoes, while negatively correlated with milk, seafood, some fruits, and starchy vegetable consumption. We otherwise refer to these patterns as the “High Alcohol & Added Sugar” and “Meat & Potatoes” patterns, respectively. Finally, the HEI-2015 was loaded positively by several fruit and vegetable categories, nuts, whole grains, and high n-3 seafood, and negatively by several meat categories, solid fats, refined grains, and added sugars. HEI-2015 was negatively correlated with all three other patterns but most strongly with Pattern #1. On average, food insecure survivors had significantly higher scores on Patterns #1 and #2 than food secure survivors (Table 3). Food insecure survivors also had significantly lower HEI-2015 scores compared to food secure survivors.

**Table 2:** Pearson correlation coefficients showing the contributions of each food group to the extracted dietary patterns

Pattern	Pattern # 1 ^†a^	Pattern #2 ^‡b^	Pattern #3 ^‡c^	HEI-2015^d^
Food groups				
Processed meats	0.080	− 0.16	**0.33**	− 0.19
Other meats^e^	0.060	− 0.090	**0.29**	− 0.12
Poultry	− 0.030	0.15	− 0.14	0.060
Seafood—High n-3	− 0.13	− 0.050	− 0.19	**0.22**
Seafood—Low n-3	− 0.050	0.030	**− 0.38**	0.16
Eggs	0.010	− 0.14	0.15	− 0.060
Solid Fats	**0.20**	− 0.11	0.080	**− 0.45**
Oils	**− 0.35**	**− 0.24**	0.12	**0.24**
Milk	0.00	− 0.040	**− 0.47**	0.16
Yogurt	− 0.080	0.13	− 0.040	0.18
Cheese	− 0.040	**− 0.29**	0.12	− 0.15
Alcohol	− 0.030	**0.48**	**0.20**	− 0.030
Fruit—Other	**− 0.37**	− 0.060	− 0.19	**0.34**
Fruit—Citrus, melons, and berries	− 0.19	− 0.11	**− 0.43**	**0.30**
Tomatoes	− 0.12	**− 0.38**	0.00	0.10
Dark-Green Vegetables	**− 0.23**	**− 0.23**	0.18	**0.32**
Dark-Yellow Vegetables	**− 0.36**	**− 0.28**	0.030	0.19
Other Vegetables	**− 0.38**	**− 0.38**	− 0.12	**0.24**
Potatoes	0.030	− 0.19	**0.24**	− 0.020
Other Starchy Vegetables	− 0.030	− 0.020	**− 0.28**	0.020
Legumes	− 0.040	0.010	0.11	0.14
Soy	− 0.12	− 0.010	0.15	0.12
Refined Grains	0.11	− 0.12	− 0.020	**− 0.36**
Whole Grains	**− 0.20**	− 0.10	− 0.11	**0.45**
Nuts	**− 0.49**	− 0.090	− 0.090	**0.38**
Added Sugars	**0.71**	**0.23**	0.030	**− 0.40**
Pattern # 1^†a^	–			
Pattern #2^‡b^	**0.40**	–		
Pattern #3^‡c^	0.090	− 0.080	–	
HEI-2015^d^	**− 0.64**	− 0.19	**− 0.25**	–

Correlations amongst the dietary patterns themselves are included at the bottom of the table in the lower triangular matrix form

Correlation coefficients (*r*) ≥ |0.20| are bolded to ease the identification of notable food groups characterizing the different patterns

This correlation analysis was performed on the testing sample described in the main text (*n* = 1,745)

Subjects were weighted, and the analysis was performed according to NCHS guidelines. All dietary patterns extraction procedures were performed on the training subsample described in the main text (*n* = 748)

^a^The high fat and sugar, low vegetable pattern

^b^The high alcohol and added sugar pattern

^c^The meat and potatoes pattern

^d^Healthy Eating Index 2015

^e^Includes red meats and organ meats

^†^Dietary pattern obtained using penalized logistic regression

^‡^Dietary pattern obtained using principal components analysis

**Table 3:** Means and standard deviations of the extracted dietary patterns across levels of food insecurity status among all cancer survivors in the testing subsample

Dietary pattern	Combined sample (*n* = 1,745)	Food-insecure (*n* = 213)	Food-secure (*n* = 1,532)	Cohen’s *d*	*p*
Pattern #1^†a^ Mean (SD)	− 0.02 (0.17)	0.07 (0.20)	− 0.03 (0.16)	0.23	< 0.01
Pattern #2^‡b^ Mean (SD)	− 0.03 (0.63)	0.10 (0.52)	− 0.04 (0.64)	0.18	< 0.01
Pattern #3^‡c^ Mean (SD)	0.13 (0.91)	0.23 (0.88)	0.12 (0.92)	0.12	0.10
HEI-2015^d^ Mean (SD)	55.13 (14.3)	50.84 (12.44)	55.61 (14.42)	− 1.30	< 0.01

Subjects were weighted, and the analysis was performed according to NCHS guidelines. All dietary patterns extraction procedures were performed on the training subsample described in the main text (*n* = 748)

^a^The high fat and sugar, low vegetable pattern

^b^The high alcohol and added sugar pattern

^c^The meat and potatoes pattern

^d^Healthy Eating Index 2015

^†^Dietary pattern obtained using penalized logistic regression

^‡^Dietary pattern obtained using principal components analysis

*p-*values are for survey-weighted *t*-tests comparing food-secure and insecure survivors from the testing subsample described in the main text (*n* = 1,745)

**Fig. 3:**
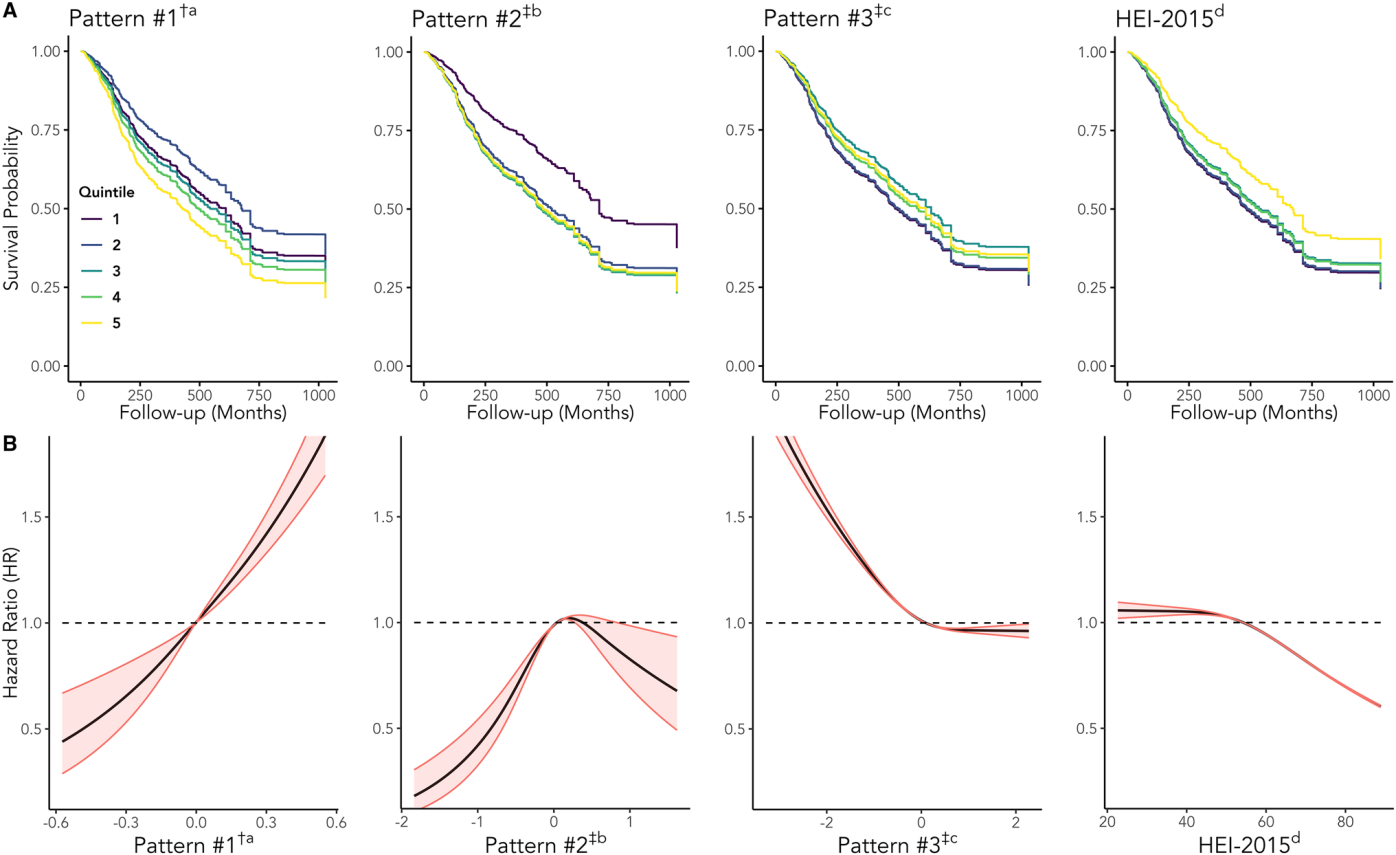
**A** Survival curves detailing the relationships between the dietary patterns and all-cause mortality among all cancer survivors in the testing subsample (*n* = 1,745). Adjusted survival curves were generated from models specified with quintile dummy variables. **B** Spline curves from expanding the diet quality index using a basis expansion for a natural cubic spline with one interior knot (see supplementary methods file for further details). The dashed line shows HR = 1, included for reference. All models adjusted for age, sex, race and ethnicity, BMI, household size, family income-to-poverty ratio, education status, health insurance status, alcohol intake, smoking status, calories, weekly MET minutes, the Charlson Comorbidity Index score, receipt of SNAP benefits, and food insecurity status*.* ^a^The High Fat & Sugar, Low Vegetable Pattern. ^b^The High Alcohol & Added Sugar Pattern. ^c^The Meat & Potatoes Pattern. ^d^Healthy Eating Index 2015. ^**†**^Dietary pattern obtained using penalized logistic regression; ^**‡**^Dietary pattern obtained using principal components analysis (PCA). Subjects were weighted, and the analysis was performed according to NCHS guidelines. All dietary patterns extraction procedures were performed on the training subsample described in the main text (*n* = 748).

In our primary analysis, and after multivariable adjustment, we found significant associations between the extracted dietary patterns and mortality (Table 4 and Supplementary Table 1). Among the testing subsample of all cancer survivors (*n* = 1,745), the highest quintile of Pattern #1 (The High Fat & Sugar, Low Vegetable Pattern) had a 1.53-fold greater risk of all-cause mortality than the lowest quintile and a standard deviation increase in the index score was associated with a 25% increased risk of all-cause mortality (in the *Full* model—Table 4). There was a similar positive, albeit weaker, association between Pattern #2 (The High Alcohol & Added Sugar Pattern) and all-cause mortality, but unlike Pattern #1 the relationship did not appear to follow a linear or dose-dependent pattern (*p*_non-linear_ < 0.01; Fig. 3). We found an inverse dose-dependent relationship between the HEI-2015 and all-cause mortality, where a standard deviation increase in the HEI-2015 score was associated with a 12% reduced risk of all-cause mortality (Table 4; Fig. 3). Among food insecure cancer survivors only (*n* = 213), there was a 47% reduced risk of all-cause mortality for every standard deviation increase in the HEI-2015 score (Supplementary Table 1). The hazard ratios for Pattern #1 for the *Full* model were similar to those from the analysis involving all cancer survivors, although they were nonsignificant and had higher variances (Supplementary Table 1).

**Table 4:** Adjusted hazard ratios (HRs) and 95% confidence intervals from the *Null*^h^, *Basic*^i^, and *Full*^j^ models for the risks of all-cause and cause-specific mortalities, in relation to the dietary patterns, in the testing subsample (*n* = 1745)

Dietary pattern	Model	Q1	Q2	Q3	Q4	Q5	*p*^a^_trend_	HR^b^_continuous_	*p*^c^_non-linear_
All-cause mortality									
Pattern #1^†d^	Null	1.00	0.79 (0.50–1.24)	1.13 (0.77–1.66)	1.34 (0.91–1.97)	1.28 (0.80–2.03)	0.08	1.14 (1.01–1.29)*	0.09
	Basic	1.00	0.88 (0.54–1.42)	1.23 (0.84–1.82)	1.45 (1.01–2.07)*	1.97 (1.24–3.13)**	< 0.01**	1.36 (1.17–1.59)**	0.63
	Full	1.00	0.75 (0.44–1.25)	1.08 (0.72–1.62)	1.23 (0.82–1.85)	1.53 (1.00–2.35)*	0.02*	1.25 (1.09–1.43)**	0.49
Pattern #2^‡e^	Null	1.00	1.49 (0.98–2.26)	1.78 (1.12–2.82)*	1.49 (0.94–2.37)	1.82 (1.16–2.85)**	0.01*	1.14 (1.02–1.27)*	0.02*
	Basic	1.00	1.84 (1.20–2.82)**	2.10 (1.35–3.27)**	2.01 (1.26–3.21)**	2.11 (1.29–3.43)**	< 0.01**	1.20 (1.06–1.35)**	< 0.01**
	Full	1.00	1.84 (1.17–2.91)**	2.06 (1.27–3.34)**	2.03 (1.21–3.41)**	1.98 (1.17–3.35)*	< 0.01**	1.15 (1.01–1.31)*	< 0.01**
Pattern #3^‡f^	Null	1.00	0.91 (0.63–1.32)	0.69 (0.47–1.01)	0.63 (0.37–1.05)	0.71 (0.46–1.07)	0.04*	0.83 (0.71–0.97)*	0.41
	Basic	1.00	0.94 (0.67–1.32)	0.77 (0.52–1.14)	0.91 (0.56–1.48)	0.86 (0.56–1.31)	0.43	0.91 (0.78–1.07)	0.30
	Full	1.00	0.98 (0.68–1.42)	0.72 (0.47–1.10)	0.83 (0.53–1.32)	0.80 (0.52–1.22)	0.22	0.88 (0.75–1.02)	0.38
HEI–2015^g^	Null	1.00	1.15 (0.71–1.86)	0.88 (0.57–1.37)	1.02 (0.64–1.63)	0.74 (0.45–1.21)	0.14	0.93 (0.83–1.05)	0.13
	Basic	1.00	0.89 (0.56–1.42)	0.77 (0.51–1.16)	0.74 (0.49–1.11)	0.49 (0.30–0.79)**	< 0.01**	0.81 (0.72–0.92)**	0.34
	Full	1.00	0.98 (0.64–1.51)	0.87 (0.60–1.26)	0.88 (0.62–1.27)	0.62 (0.39–0.97)*	0.02*	0.88 (0.78–0.99)*	0.48
Cancer-specific mortality									
Pattern #1^†d^	Null	1.00	0.87 (0.44–1.72)	0.82 (0.46–1.46)	1.30 (0.67–2.54)	1.93 (0.92–4.05)	0.08	1.27 (1.03–1.57)*	0.58
	Basic	1.00	0.98 (0.49–1.99)	0.93 (0.51–1.67)	1.38 (0.70–2.71)	2.77 (1.33–5.80)**	0.02*	1.50 (1.15–1.95)**	0.88
	Full	1.00	0.88 (0.43–1.80)	0.92 (0.51–1.68)	1.35 (0.67–2.73)	2.23 (1.31–3.78)**	< 0.01**	1.36 (1.13–1.63)**	0.88
Pattern #2^‡e^	Null	1.00	0.99 (0.54–1.84)	1.78 (0.97–3.29)	1.39 (0.70–2.78)	2.70 (1.36–5.37)**	< 0.01**	1.24 (1.07–1.44)**	0.10
	Basic	1.00	1.27 (0.65–2.46)	2.08 (1.10–3.94)*	1.84 (0.87–3.89)	2.95 (1.40–6.22)**	< 0.01**	1.26 (1.09–1.46)**	0.04*
	Full	1.00	1.21 (0.62–2.36)	2.12 (1.07–4.20)*	1.72 (0.81–3.65)	2.23 (1.15–4.35)*	0.01*	1.16 (1.00–1.34)*	0.08
Pattern #3^‡f^	Null	1.00	0.64 (0.39–1.06)	0.53 (0.30–0.92)*	0.74 (0.31–1.80)	0.78 (0.45–1.37)	0.58	0.95 (0.70–1.29)	0.04*
	Basic	1.00	0.70 (0.43–1.13)	0.60 (0.33–1.12)	1.04 (0.45–2.44)	0.91 (0.54–1.54)	0.95	1.02 (0.78–1.34)	0.06
	Full	1.00	0.73 (0.44–1.21)	0.63 (0.33–1.20)	0.84 (0.50–1.41)	0.84 (0.50–1.40)	0.59	0.96 (0.76–1.22)	0.12
HEI-2015^g^	Null	1.00	0.88 (0.33–2.37)	0.48 (0.22–1.05)	0.88 (0.39–1.98)	0.50 (0.22–1.17)	0.15	0.88 (0.71–1.08)	0.42
	Basic	1.00	0.71 (0.27–1.85)	0.43 (0.20–0.92)*	0.69 (0.33–1.44)	0.37 (0.17–0.83)*	0.02*	0.79 (0.65–0.97)*	0.69
	Full	1.00	0.81 (0.38–1.74)	0.52 (0.30–0.89)*	0.84 (0.53–1.35)	0.44 (0.25–0.77)**	< 0.01**	0.83 (0.70–0.97)*	0.75

Subjects were weighted, and the analysis was performed according to NCHS guidelines

This survival analysis was performed on the testing subsample described in the main text (*n* = 1,745). All dietary patterns extraction procedures were performed on the training subsample described in the main text (*n* = 748)

^a^Test for trend across the quintiles of the dietary exposure. See Eq. 2 in the main text

^b^Hazard ratio for a standard deviation increase in the dietary exposure variable. See Eq. 3 in the main text

^c^Likelihood ratio test *p*-value for a natural cubic spline model (Eq. 4 in the main text) compared to specifying the model with the scaled dietary exposure variable (Eq. 3)

^d^The high fat and sugar, low vegetable pattern

^e^The high alcohol and added sugar pattern

^f^The meat & potatoes pattern

^g^Healthy Eating Index 2015

^h^Includes the dietary pattern score variable with no additional covariates

^i^Further adjusts for age, sex, and race and ethnicity

^j^Further adjusts for BMI, household size, family income-to-poverty ratio, education status, health insurance status, receipt of SNAP benefits, food insecurity status, alcohol intake, smoking status, total caloric intake, weekly MET minutes, and the Charlson Comorbidity Index score

^**†**^Dietary pattern obtained using penalized logistic regression

^**‡**^Dietary pattern obtained using principal components analysis (PCA)

^**^p < 0.01; *p < 0.05

When we examined cancer-specific mortality, the parameter estimates for all cancer survivors in the testing subsample were similar to those for all-cause mortality (Table 4). We observed a positive association between Pattern #1 and cancer-specific mortality, where a standard deviation increase in the score was associated with a 36% increased risk of cancer mortality. The HEI-2015 was again inversely associated with cancer mortality, where a standard deviation increase in the score was associated with a 17% reduced cancer-related mortality risk. A significant and robust positive association was also observed between Pattern #2 and cancer-related mortality. When the analysis was restricted to food insecure cancer survivors in the testing subsample, we found that the hazard ratios mirrored those from the analysis of all cancer survivors combined, though they were not statistically significant (Supplementary Table 1). Further adjustment for the NHANES ADL score (Supplementary Table 2) did not significantly alter the parameter estimates though there was only a significant association between Pattern #1 and all-cause mortality. Additionally, we found significant and positive associations involving Patterns #1 and #3 when including only participants with a primary cancer diagnosis within the four years before their study interview (Supplementary Table 3). In the propensity score matching analysis (Supplementary Tables 4, 5), we found significant associations involving Patterns #1 and #2 and all-cause mortality among all cancer survivors. The hazard ratios in these cases were similar in magnitude and direction to those we observed in the main analysis. We also found a significant inverse relationship between Pattern #3 and cancer-specific mortality among food insecure cancer survivors that was not consistent with any previous analysis.

## Discussion

Using a nationally representative sample of U.S. cancer survivors, we found that dietary patterns associated with being a food insecure cancer survivor were positively associated with all-cause and cancer-specific mortality after adjusting for several confounders. Of the three dietary patterns we extracted from the observed 24-h recall data (one with penalized logit and two with PCA), two of these patterns—Pattern #1 and Pattern #2, which were both loaded by high consumption of palatable and processed foods and low loadings of fruits, vegetables, and other healthy components—were robustly and positively associated with all-cause and cancer-specific mortalities among cancer survivors and a subset of food insecure cancer survivors. However, Pattern #1 exhibited the most robust set of associations across all sensitivity and subanalyses. Finally, the validity of the extracted dietary patterns was supported by comparison to the HEI-2015, which indicated lower diet quality (with respect to adherence to the Dietary Guidelines for Americans 2015) in food insecure survivors compared to food secure survivors and which was significantly and inversely correlated with the extracted dietary patterns.

Our findings contribute to evidence highlighting the adverse associations between food insecurity, dietary behaviors, and health outcomes. However, our work is novel in that we focused on cancer survivors, a population that has received relatively little scrutiny within the broader context of food insecurity despite the fact that this population may have an elevated risk of experiencing food insecurity [3, 24, 25]. Several lines of evidence tie food insecurity to an increased comorbidity burden, including increased risks of hypertension, hyperlipidemia, diabetes, and mental health conditions [5, 26, 27]. Moreover, food insecurity is associated with poor overall health status, and recent analyses using NHANES data demonstrated significant and positive associations between food insecurity status and the risk of all-cause and cardiovascular disease mortality [23, 28–31]. Our analysis complements this body of work by demonstrating that dietary intake may be pertinent to the pathway between food insecurity and increased mortality in cancer survivors. However, mediation analyses are needed to support this conjecture.

Diet quality is associated with physiological outcomes that may help explain the differential propensity for survival, as observed in our analysis. In a longitudinal sample of older adults from the Health and Retirement Study, higher diet quality, as measured by the HEI-2015, was associated with better lipid and C-reactive protein (CRP) profiles and decreased likelihood of depression and functional deficits [32]. In another longitudinal sample from the Health, Eating, Activity, and Lifestyle (HEAL) prospective cohort study, higher postdiagnosis HEI-2015 scores were associated with lower CRP levels in breast cancer survivors [33]. A nested cross-sectional study from the Multiethnic Cohort Study examined relationships between four a priori diet quality indices (AHEI-2010, HEI-2010, aMED, and DASH) and several serum carotenoids and biomarkers (leptin, HOMA-IR, glucose, CRP, insulin, and triglycerides) and found that higher diet index scores were positively associated with carotenoid markers and inversely associated with other biomarkers [34]. Finally, in a cross-sectional analysis of newly diagnosed head and neck squamous cell carcinoma patients, higher diet quality, measured by a “whole foods” dietary pattern extracted using PCA from FFQ data, was inversely associated with several pro-inflammatory cytokines [35]. Thus, the link between diet quality and downstream inflammation may explain our observed results, particularly in the context of cancer, where higher inflammatory biomarkers exacerbate disease progression, resulting in a poor prognosis [36–38].

Our findings have policy implications. As alluded to in our previous analysis, screening for food insecurity is not a clinical best practice widely implemented in cancer clinics, although the National Comprehensive Cancer Network recently incorporated an item dedicated to food insecurity in its Distress Thermometer screener [3, 11, 39]. Identifying food insecure patients in the oncology care setting, early in the cancer care continuum and later as care progresses and financial hardship may be exacerbated, can facilitate prompt referral to additional support resources. These resources could be accessed, for example, through a case manager or social worker who assists the cancer survivor in leveraging personal and community-level resources or providing referrals to federal and local nutrition assistance programs [3, 40]. The establishment of hospital-based food pantries is another avenue that has shown promise for cancer survivors to access nutritious foods they may otherwise lack access to [41]. Thus, tailoring community- and higher-level initiatives prioritizing food support throughout the treatment phase and the prolonged post-treatment phase may be critical to mitigating the negative health consequences that food insecure cancer survivors may experience secondary to the lack of a steady stream of nutritious foods [42].

This analysis has several strengths, including the nationally representative sample from the NHANES, the use of a validated food insecurity measurement tool, the quality and quantity of covariate data used to account for potential and known confounders, and the quality of the linked mortality data through the NCHS Linked Mortality Files. Nevertheless, there are limitations to note. First, we used a static measure of dietary intake. However, we know that dietary intake patterns are dynamic and circumstantial, and our analysis could not account for any variation in dietary intake over time despite using time-to-event measures that occurred substantially after the dietary intake measurement instance. Similarly, it is worth considering that food insecurity can be a transient phenomenon that subjects recover from, which may have occurred for participants in the intervening window between the study visit and the time of the observed event or censoring. An additional consideration concerning the measurement of dietary intake using 24-h recalls is that it may be subject to systematic measurement errors that we could not quantify with the available data. We must also qualify that our findings are based on a set of 24-h recalls, which are not designed to capture and may not accurately represent long-term dietary intake, unlike other measurement tools such as FFQs. However, we acknowledge that these data are the best we currently have for answering our research questions in the setting of a large epidemiological survey study. Second, with any analysis of observational data outside of a rigid set of assumptions, we must conclude that unmeasured or residual confounding cannot be excluded and that no causal interpretations should be made with these results. Tumor staging may have confounded the results, but we could not control for this given that these data are not collected in either the NHANES survey or examination. Similarly, we were not able to control for tumor site. Third, although we did not account for stress as a confounding variable, given limitations with measures of psychological stress or allostatic load in the NHANES survey and our sample size, we believe it is appropriate to conclude that measures of food insecurity, such as those captured by the USDA FSSM, are likely to be highly correlated with measures of stress. Indeed, the U.S. Household FSSM includes questions designed to capture concern and stress about food insufficiency, such as: (*I/We*)* worried whether *(*my/our*)* food would run out before *(*I/we*)* got money to buy more* [43]. Fourth, selection bias may have occurred in the recruitment of cancer survivors into the NHANES study (e.g., survivors with more advanced cancers or with specific cancer types may have exhibited lower response rates). However, any bias is conjectural given the lack of cancer stage or other clinical data in the NHANES to make any conclusions on this type of bias and should be kept in mind when generating conclusions from our results. Fifth, smoking behaviors were significantly more prevalent among food insecure cancer survivors compared to their food secure counterparts. Although we adjusted for smoking behavior in our analyses, there may be residual confounding related to smoking status. Finally, a critical reflection of using the U.S. Household FSSM is that a measure of household food insecurity may not capture the burden of food insecurity exacted on any individual within that household. It is also imperative to qualify that the dietary patterns extracted in this analysis reflect population-level summary measures of dietary intake and should not be used to make conclusions about dietary intake for an individual cancer survivor experiencing food insecurity.

In summary, we conclude that dietary patterns extracted with empirical methods, used to characterize the overall dietary behaviors of U.S. food insecure cancer survivors, may deleteriously impact cancer-related outcomes such as all-cause and cancer-specific mortality. These patterns, characterized by the consumption of added sugars and processed foods with concomitant low consumption of fruits, vegetables, whole grains, and other healthy diet components, highlight an urgent public health challenge demanding innovative policy and community-level solutions. We identified several avenues for future research in this area. One avenue includes developing and evaluating community- and individual-level interventions for bolstering food security among food insecure cancer survivors throughout the early treatment and cost-prohibitive phases of the cancer care continuum. A second avenue should focus on piloting interventions for medical provider training in screening for food insecurity in oncology settings. A third avenue should implement this analysis in other large survey studies to gauge the reproducibility of these dietary patterns within this target population. A final avenue of research should extend our work and continue surveillance of dietary intake patterns amongst U.S. food insecure cancer survivors using nationally representative data. Ultimately, advances in such areas will ideally abate the disparities in health outcomes observed by food insecure cancer survivors, highlighted by our work and other colleagues.

### Supplementary Information

Below is the link to the electronic supplementary material.

Supplementary file1 (PDF 231 KB)

Supplementary file2 (PDF 256 KB)

## Data Availability

The datasets generated during and/or analyzed during the current study are available in a public GitHub repository, https://github.com/cmainov/nhanes-fi-ca-mortality-mirror.
