# Supplementary material for: Dietary patterns among U.S. food insecure cancer survivors and the risk of mortality: NHANES 1999–2018
Source: Cancer Causes Control. 2024 Mar 26;35(7):1075–88. doi: 10.1007/s10552-024-01868-2 (PMC11217055; doi:10.1007/s10552-024-01868-2)
Supplement: Supplementary file 1 — Supplementary file1 (PDF 231 KB) [file 10552_2024_1868_MOESM1_ESM.pdf]

Supplementary Methods

Christian A. Maino Vieytes, Ruoqing Zhu

Contents

1 Dietary Patterns Extraction with Penalized Logit 1
1.1 Rationale 1
1.2 Implementation 2
1.3 Dependent and Explanatory Variables 2
1.4 Implementation in R 2

2 Cubic Splines 3
2.1 Background 3
2.2 Basis Representation for Cubic Splines 3
2.3 Natural Cubic Splines 4
2.4 Implementation in R 5

3 Propensity Score Matching 7

1 Dietary Patterns Extraction with Penalized Logit

1.1 Rationale

We implemented penalized logistic regression with an elastic net penalty to extract dietary patterns from observed dietary intake data from 24-hour recalls as explained in the main text. The theory and rationale for the elastic net is proposed by Zou and Hastie.[1] The idea involves having a regularization technique that accomplishes both *shrinkage* and *variable selection* simultaneously. The goal was to extract dietary patterns associated with a given binary characteristic. We chose the elastic net given the high correlation amongst dietary variables in epidemiologic studies and the unique ability of elastic net to handle correlated variables. Unlike ridge regression, elastic net allows us to achieve parsimony and also avoids the sparsity that often accompanies the LASSO model.[1]

1.2 Implementation

The elastic net algorithm begins by the specification of a logistic regression model with the elastic net penalty, which is often conceptualized as a mixture of the LASSO penalty (ℓ_1) and ridge regression penalty (ℓ_2) terms. Equation 1 demonstrates the formulation of the objective function. The penalty term introduced for the logistic regression case is introduced into the likelihood function used to fit the model:

$$-2[\ell(\beta)] = -2\sum_{i=1}^{n} log(Pr(Y_i|X_i,\beta) + \lambda[(1-\alpha)]||\beta||_2^2 + \alpha||\beta||_1]]) \quad (1)$$

The -2log-likelihood includes the -2log-likelihood function for a logistic regression model plus an additional penalty term, $\lambda[(1-\alpha)]||\beta||_2^2 + \alpha||\beta||_1]]$, which involves a combination of the (ℓ_1) and (ℓ_2) penalities and two tuning parameters, λ and α, which are unknown and conventionally optimized via K-fold cross-validation. Optimizing this objective function relies on numerical methods.[2] We used 10-fold cross-validation and minimized the deviance to find the optimal combination of λ and α. A grid of α values from 0 to 1 in 0.1 increments was used.

1.3 Dependent and Explanatory Variables

As indicated in the main text, we selected the binary household food insecurity status (constructed using variable `FSDHH` in the NHANES dataset) for the penalized logitic regression model. The explanatory variables were intake equivalents for 26 food group variables from the USDA Food Patterns Equivalents Database (FPED) and the MyPyramid Equivalents Database (MPED) data. The exact variables are provided in the main text in Table 2. All food group variables were centered and scaled (by their standard deviation) prior to fitting the elastic net models. We also included total calories (not scaled or centered) in the model to adjust for total energy intake via the standard multivariate method.[3] All data wrangling and management steps done prior to the analysis are provided in the following file: https://github.com/cmainov/nhanes-fi-ca-mortality/blob/main/R/01-covariate-mortality-data-linkage.R.

1.4 Implementation in R

The `glmnet` and `caret` packages in `R` can be used to fit models with elastic net, LASSO, and ridge penalization. R code to extract the dietary patterns is available in the following files:

(i) https://github.com/cmainov/nhanes-fi-ca-mortality/blob/main/R/03-diet-score-computations.R (*All steps going from data import to outputting a dataset with the diet pattern scores*)

(ii) https://github.com/cmainov/nhanes-fi-ca-mortality/blob/main/R/utils.R (*Helper functions written for use in the above script*)

2 Cubic Splines

2.1 Background

Using splines in regression models is a popular method for flexibly modeling exposure-outcome relationships in epidemiological studies.[4,5] Splines in a regression context can be conceptualized as a series of local polynomials fit over the domain of the regression function. It provides a nice alternative to a categorical dose-response analysis, which has several limitations. One of those key limitations is the assumption that the response is uniform over all observations within a category for which a parameter estimate is made, resulting in the appearance of a step-wise function for the relationship between exposure and response.[6] In contrast, fitting a model with spline terms is a parametric technique that generates a smooth curve, allowing the user to visualize the dose-response relationship. The parameter estimates are rarely of interest owing to their lack of interpretability.[6] However, given that a model specifying the exposure as a continuous variable is nested in a model with an expansion of spline terms for that variable, the departure from a linear relationship can be formally tested with a Likelihood Ratio Test.[5]

2.2 Basis Representation for Cubic Splines

Fitting spline models involves applying a set of transformations to the exposure variable, X, including them in an additive model, and estimating parameters as is conventional in regression. In the regression model, we replace the original variable with the set of transformations (which we term *basis functions*) and estimate parameters for each of those transformations:

$$E[Y|X,V] = \sum_{m=1}^{M} \beta_m h_m(X) + \gamma V$$

which is a linear basis expansion in X, where h_m is the mth basis function (of which there are M), and γV is a term for an additional covariate we may want to adjust for (no basis expansion on this term).[7] We generalize this approach to the multivariable setting where we desire basis expansions in several variables:

$$E[Y|X,V] = \sum_{j=1}^{J} \sum_{m=1}^{M_j} \beta_{jm} h_{jm}(X_j) + \gamma V$$

where j is the index of variables. The set of fixed basis functions is of interest, as are the *knots* and *knot locations*, which are the regions along the domain where

separate local piecewise polynomials are fit. Uniform or non-uniform spacing can be used to specify the knots, although a set of quantiles of the exposure are conventionally used as the knot locations.[8] To achieve smoothness in the spline curve across these disjoint regions, a continuity constraint is applied. Using a truncated power basis, we achieve the continuity constraint:

$$(x-\xi_\ell)_+^g = \begin{cases} (x-\xi_\ell)^g, & \text{if } x > \xi_\ell \\ 0, & otherwise \end{cases}$$

where ξ_ℓ is the ℓth knot and g is the order of the polynomial we choose to fit. We arrive at the number of basis functions by beginning with basis functions for the degree of the polynomial we desire and then include one truncated power basis function for each knot we specify (an additional basis function where we apply a constant–i.e., 1–can also be included for the intercept).[8] For a cubic spline (where $g = 3$) with two interior knots, we use the following basis functions:

$$h_1(x) = 1, h_2(x) = x, h_3(x) = x^2, h_4(x) = x^3, h_5(x) = (x-\xi_1)_+^3, h_6(x) = (x-\xi_2)_+^3$$

It is shown that for a degree g polynomial, the basis functions will results in a continuous curve (i.e., smooth) up to the $(g-1)$ derivative.[8] For the cubic spline basis representation above, we have that Y will be continuous up to its second derivative at each knot boundary. For a cubic spline, we determine the degrees of freedom by counting the number of parameters we must estimate within each interval and the number of constraints. That is, we require 4 parameters within each interval and 3 constraints (continuity in Y, Y', and Y'') at each knot. If we specify two interior knots ($K = 2$) we have:

$$4(K+1) - 3K = 4(2+1) - (3)(2) = 6 \ df$$

and $K+1 = 2+1 = 3$ intervals. We can fit the model using standard statistical software, and estimation is carried out in a routine manner (e.g., least squares for linear regression or maximum likelihood estimation for generalized linear models).

2.3 Natural Cubic Splines

A cited limitation of cubic splines is the high variance in the spline estimates at the extremes of the data (i.e., in the highest and lowest intervals).[7] Natural cubic splines take cubic splines and apply two additional constraints: that the function is linear beyond the boundary knots (i.e., the exterior knots–if we have two interior knots, then we have two additional exterior or boundary knots). Y'' and Y''' will be zero at these knot boundaries, and we will have K basis functions for a natural cubic spline with K knots (K here assumes we

count the two exterior or boundary knots in addition to the interior knots–for a spline with two interior knots we have $K = 4$).

In our analysis, we specify the diet quality indices using a basis expansion for a natural cubic spline with one interior knot ($K = 3$). These basis functions are provided below.

In a general form[7] :

$$h_1(x) = 1, h_2(x) = x, h_{k+2}(x) = d_k(x) - d_{K-1}(x)$$
$$\text{where } d_k(x) = \frac{(x - \xi_k)^3_+ - (x - \xi_K)^3_+}{\xi_K - \xi_k} \text{ and } k \in \{1...K-2\}$$

And in the case of $K = 3$:

$$h_1(x) = 1, h_2(x) = x, h_3(x) = d_1(x) - d_2(x)$$

2.4 Implementation in R

Natural cubic splines are implemented in R using the `ns()` function from the `splines` package. The R code we specified for this analysis is found at: https://github.com/cmainov/nhanes-fi-ca-mortality/blob/main/R/utils.R. We demonstrate the use of `ns()` with a simple, reproducible example. First, we generate some data by sampling from two Gaussian distributions and then use the `ns()` function to generate a matrix with the basis functions in its columns. We want to use *two* interior knots ($K = 4$), so we will specify the `df = 3` argument. `ns()` counts the interior knots in the following way:

$$K_{interior} = df - intercept - 1$$

where $intercept \in \{0, 1\}$ and is controlled by the `intercept =` argument in the `ns()` function. Note: the default value is `intercept = FALSE`, which results in $intercept = 0$. The `lm()` function will compute the intercept for us, and, thus, there is no need to redundantly specify it within `ns()`

```
# generate some toy data
set.seed( 23 ) # set seed for reproducibility
x <- rnorm( 20, mean = 35, sd = 5 )
y <- rnorm( 20, mean = 78, sd = 10 )
```

```
# specify a natural cubic spline with 2 interior knots (K=4)
# (generate the basis matrix)
k2 <- ns( x, df = 3 )

head( k2, 5 ) # print first 5 rows of the basis matrix

# 1          2           3
# [1,]  0.27013521 0.4949742 -0.31550699
# [2,] -0.07878135 0.4987137 -0.33410946
# [3,]  0.53390462 0.3255878  0.03408727
# [4,] -0.14194707 0.4300676  0.71187952
# [5,]  0.50852945 0.3222180  0.09029069
```

We then can use this matrix and specify a linear regression model, regressing `y` on the basis expansion in `x`:

```
# linear regression model with 'ns'
lm( y ~ ns( x, df = 3 ) )

# Call:
#   lm(formula = y ~ ns(x, df = 3))
#
# Coefficients:
#   (Intercept)  ns(x, df = 3)1  ns(x, df = 3)2  ns(x, df = 3)3
# 80.999           6.804          -13.666           -2.166
```

The output reflects the parameter estimates for the four basis functions we detailed above (including one for the intercept).

Note: we can supply R with the exact locations of the interior and boundary knots and get the same basis matrix (instead of specifying the `df` argument– note there might be a slight rounding error in this matrix compared to the one above).

```
# alternative code for specifying the same matrix
k2.v2 <- ns( x,
    knots = quantile( x, c( 0.33, 0.66 ) ), # interior knots (1st and 2nd tertiles)
    Boundary.knots = quantile( x, c( 0, 1 ) ) ) # boundary knots (min and max)

head( k2.v2, 5 ) # print first 5 rows of the basis matrix

# 1          2           3
# [1,]  0.27454544 0.4970633 -0.31053853
# [2,] -0.08486486 0.5069896 -0.33380781
# [3,]  0.52668014 0.3284054  0.04392163
# [4,] -0.14508287 0.4247300  0.72035287
```

```
# [5,]  0.49944729 0.3253491  0.10026887
```

Final notes: (i) the `ns()` implements a slightly modified version of the basis functions for the natural cubic spline (which is why we do not get the exact values in the basis matrix that we would have otherwise predicted). Indeed, `ns()` implements a linear transformation of the basis to generate a B-spline basis matrix for the natural cubic spline we seek to fit (done for computational reasons). (ii) We note that an extension of the natural cubic spline approach we present would be to use *smoothing splines*, which we did not use in our analysis and that allow for a more objective conclusion on the number of knots to assign to the data.[7]

3 Propensity Score Matching

As described in the main text, we conducted a sensitivity analysis whereby the analytic sample was refined based on matched samples in high and low fractions of the dietary patterns scores with similar covariate values.[9] Specifically, we used weighted logistic regression model to estimate the probability of having a high dietary pattern score ("high" was defined as having a dietary pattern index score $\geq$ the median value). We then assessed for overlap in the propensity scores across "high" and "low" groups of the diet scores (i.e., "common support"), effectively removing subjects from the sample that lacked a potential match (based on the similarity of the propensity score).[10] Finally, Nearest Neighbor matching was used to assign matches in the "high" and "low" groups of the diet scores.[11] Propensity score estimation and the matching steps were implemented with the `matchit` function from the `MatchIt` R package.[12] Because no software for this type of procedure accounts for all aspects of the survey design, we used normalized weights in the weighted logistic regression models that estimated the propensity scores. The code for this analysis is found at: https://github.com/cmainov/nhanes-fi-ca-mortality/blob/main/R/06-ps-matching-sensitivity.R.

References

1. Zou, H. & Hastie, T. Regularization and variable selection via the elastic net. *Journal of the Royal Statistical Society Series B: Statistical Methodology* **67,** 301–320 (2005).
2. Jin, B., Lorenz, D. A. & Schiffler, S. Elastic-net regularization: error estimates and active set methods. *Inverse Problems* **25,** 115022 (2009).
3. Willett, W. C., Howe, G. R. & Kushi, L. H. Adjustment for total energy intake in epidemiologic studies. *The American journal of clinical nutrition* **65,** S1220–S1228 (1997).
4. Greenland, S. Dose-response and trend analysis in epidemiology: alternatives to categorical analysis. eng. *Epidemiology (Cambridge, Mass.)* **6,** 356–365. doi:10.1097/00001648-199507000-00005 (July 1995).

5. Witte, J. S. & Greenland, S. A nested approach to evaluating dose-response and trend. en. *Annals of Epidemiology* **7,** 188–193. doi:10.1016/S1047-2797(96)00159-7 (Apr. 1997).
6. Steenland, K. & Deddens, J. A. A Practical Guide to Dose-Response Analyses and Risk Assessment in Occupational Epidemiology. *Epidemiology* **15.** Publisher: Lippincott Williams & Wilkins, 63–70 (2004).
7. Hastie, T., Tibshirani, R. & Friedman, J. *The Elements of Statistical Learning* ISBN: 978-0-387-84857-0 978-0-387-84858-7. doi:10.1007/978-0-387-84858-7 (Springer New York, New York, NY, 2009).
8. James, G., Witten, D., Hastie, T. & Tibshirani, R. *An Introduction to Statistical Learning* ISBN: 978-1-4614-7137-0 978-1-4614-7138-7. doi:10.1007/978-1-4614-7138-7 (Springer New York, New York, NY, 2013).
9. Austin, P. C. The relative ability of different propensity score methods to balance measured covariates between treated and untreated subjects in observational studies. *Medical decision making* **29,** 661–677 (2009).
10. Garrido, M. M. *et al.* Methods for constructing and assessing propensity scores. *Health services research* **49,** 1701–1720 (2014).
11. Austin, P. C. A comparison of 12 algorithms for matching on the propensity score. *Statistics in medicine* **33,** 1057–1069 (2014).
12. Ho, D. E., Imai, K., King, G. & Stuart, E. A. MatchIt: Nonparametric Preprocessing for Parametric Causal Inference. *Journal of Statistical Software* **42,** 1–28. doi:10.18637/jss.v042.i08 (2011).
