# Supplementary material for: Dietary patterns among U.S. food insecure cancer survivors and the risk of mortality: NHANES 1999–2018
Source: Cancer Causes Control. 2024 Mar 26;35(7):1075–88. doi: 10.1007/s10552-024-01868-2 (PMC11217055; doi:10.1007/s10552-024-01868-2)
Supplement: Supplementary file 2 — Supplementary file2 (PDF 256 KB) [file 10552_2024_1868_MOESM2_ESM.pdf]

Supplementary Table 1. Adjusted hazard ratios (HRs) and 95% confidence intervals from the *Null*[h], *Basic*[i], and *Full*[j] models for the risks of all-cause and cause-specific mortalities, in relation to the dietary patterns, within the food-insecure cancer survivor testing subsample (*n* = 213).

Dietary Pattern	Model	Q1	Q2	Q3	Q4	Q5	p^{a}_{trend}	$HR^{b}_{continuous}$	$p^{c}_{non-linear}$
All-Cause Mortality									
Pattern #1[†d]	*Null*	1.00	1.23 (0.31-4.89)	3.90 (1.15-13.29)*	2.78 (1.06-7.31)*	2.32 (0.55-9.82)	0.16	1.20 (0.92-1.55)	0.30
	Basic	1.00	2.68 (0.42-17.04)	6.15 (1.16-32.58)*	4.43 (0.82-23.97)	3.07 (0.49-19.45)	0.31	1.29 (0.95-1.74)	0.52
	Full	1.00	1.48 (0.17-12.79)	5.24 (0.72-38.15)	3.54 (0.56-22.40)	1.61 (0.22-12.02)	0.69	1.17 (0.69-1.99)	0.77
Pattern #2[‡e]	*Null*	1.00	2.55 (0.64-10.15)	2.96 (0.75-11.61)	2.04 (0.38-10.96)	1.24 (0.24-6.30)	0.86	1.17 (0.79-1.73)	0.89
	Basic	1.00	3.64 (0.61-21.86)	3.00 (0.65-13.79)	3.12 (0.38-25.50)	1.19 (0.13-10.56)	0.97	1.12 (0.72-1.76)	0.30
	Full	1.00	2.62 (0.49-13.96)	1.77 (0.35-8.79)	2.44 (0.36-16.48)	0.75 (0.14-4.02)	0.52	0.98 (0.64-1.52)	0.63
Pattern #3[‡f]	*Null*	1.00	1.44 (0.53-3.91)	0.43 (0.14-1.35)	0.63 (0.19-2.15)	0.80 (0.31-2.09)	0.32	0.93 (0.67-1.28)	0.88
	Basic	1.00	1.95 (0.50-7.68)	0.88 (0.18-4.30)	1.52 (0.33-7.07)	1.26 (0.32-4.88)	0.88	1.02 (0.70-1.48)	0.71
	Full	1.00	1.39 (0.38-5.08)	0.34 (0.09-1.30)	1.16 (0.15-9.23)	0.69 (0.14-3.43)	0.43	0.81 (0.49-1.32)	0.75
HEI-2015[g]	*Null*	1.00	0.63 (0.30-1.35)	0.46 (0.15-1.42)	0.03 (0.01-0.17)**	0.34 (0.12-0.99)*	< 0.01**	0.51 (0.33-0.78)**	0.98
	Basic	1.00	0.64 (0.32-1.25)	0.60 (0.23-1.55)	0.03 (0.00-0.18)**	0.47 (0.16-1.35)	< 0.01**	0.46 (0.29-0.72)**	0.84
	Full	1.00	0.88 (0.32-2.45)	0.50 (0.17-1.48)	0.04 (0.01-0.25)**	0.76 (0.25-2.28)	0.04*	0.53 (0.33-0.86)**	0.77
Cancer-Specific Mortality									
Pattern #1[†d]	*Null*	1.00	8.32 (0.82-84.06)	16.29 (1.44-183.83)*	7.99 (0.56-114.22)	24.06 (3.04-190.66)**	0.05*	1.36 (0.97-1.90)	0.81
	Basic	1.00	13.96 (1.45-134.72)*	17.54 (1.94-158.72)*	10.10 (0.56-183.54)	19.38 (1.89-199.01)*	0.28	1.38 (0.76-2.50)	0.88
	Full	1.00	40.30 (3.08-528.16)**	38.35 (4.02-366.23)**	5.01 (0.20-125.49)	19.51 (0.94-404.64)	0.40	1.22 (0.59-2.52)	0.58
Pattern #2[‡e]	*Null*	1.00	0.40 (0.05-3.28)	0.76 (0.12-5.06)	1.39 (0.18-10.51)	0.53 (0.07-4.24)	0.86	1.08 (0.44-2.69)	0.42
	Basic	1.00	0.20 (0.00-9.97)	0.49 (0.07-3.54)	1.00 (0.10-10.43)	0.18 (0.01-3.46)	0.49	0.86 (0.31-2.34)	0.69
	Full	1.00	0.20 (0.01-3.30)	0.13 (0.01-3.32)	0.27 (0.02-4.31)	0.10 (0.00-4.83)	0.37	0.86 (0.28-2.58)	0.54
Pattern #3[‡f]	*Null*	1.00	0.44 (0.08-2.37)	0.25 (0.05-1.35)	0.45 (0.06-3.29)	0.00 (0.00-0.01)**	0.03*	0.68 (0.49-0.95)*	0.30
	Basic	1.00	0.58 (0.09-3.79)	0.45 (0.07-3.04)	0.56 (0.10-3.12)	0.00 (0.00-0.03)**	0.04*	0.70 (0.49-1.02)	0.39
	Full	1.00	0.79 (0.13-4.60)	0.12 (0.01-1.44)	0.63 (0.04-11.16)	0.00 (0.00-0.02)**	0.07	0.29 (0.06-1.40)	0.50
HEI-2015[g]	*Null*	1.00	0.93 (0.17-5.17)	1.02 (0.17-6.32)	0.07 (0.01-0.67)*	0.08 (0.01-0.67)*	0.01*	0.54 (0.34-0.87)*	0.30
	Basic	1.00	0.99 (0.20-4.90)	1.02 (0.22-4.69)	0.06 (0.01-0.64)*	0.11 (0.02-0.52)**	< 0.01**	0.49 (0.28-0.86)*	0.50
	Full	1.00	2.01 (0.25-16.18)	1.03 (0.11-9.96)	0.07 (0.00-1.12)	0.14 (0.02-1.06)	0.09	0.60 (0.28-1.30)	0.50

** $p < 0.01$; * $p < 0.05$

[a] Test for trend across the quintiles of the dietary exposure. See Equation 2 in the main text.

[b] Hazard ratio for a standard deviation increase in the dietary exposure. See Equation 3 in the main text.

[c] Likelihood ratio test *p*-value for a natural cubic spline model (Equation 4 in the main text) compared to specifying the model with the scaled dietary exposure (Equation 3).

[d] The High Fat & Sugar, Low Vegetable Pattern; [e] The High Alcohol & Added Sugar Pattern; [f] The Meat & Potatoes Pattern; [g] Healthy Eating Index 2015

[h] Includes the dietary pattern score variable with no additional covariates.

[i] Further adjusts for age, sex, and race and ethnicity.

[j] Further adjusts for BMI, household size, family income-to-poverty ratio, education status, health insurance status, receipt of SNAP benefits, food insecurity status, alcohol intake, smoking status, total caloric intake, weekly MET minutes, and the Charlson Comorbidity Index score.

[†] Dietary pattern obtained using penalized logistic regression; [‡] Dietary pattern obtained using principal components analysis (PCA).

Subjects were weighted, and the analysis was performed according to NCHS guidelines.

This survival analysis was performed on the food-insecure subset (*n* = 213) of the testing subsample described in the main text (*n* = 1745). All dietary patterns extraction procedures were performed on the training subsample described in the main text (*n* = 748).

Supplementary Table 2. Adjusted hazard ratios (HRs) and 95% confidence intervals for the risks of all-cause and cause-specific mortalities, in relation to the dietary patterns, in the testing subsample with further adjustment for the NHANES Activities of Daily Living (ADL) score (n = 905).

**Dietary Pattern**	**Model**	**Q1**	**Q2**	**Q3**	**Q4**	**Q5**	p^{a}_{trend}	$HR^{b}_{continuous}$	$p^{c}_{non-linear}$
All-Cause Mortality									
Pattern #1[†d]	*Full*[h]	1.00	0.74 (0.41-1.35)	0.95 (0.55-1.62)	1.25 (0.72-2.16)	1.45 (0.83-2.54)	0.15	1.24 (1.02-1.51)*	0.60
Pattern #2[‡e]	*Full*[h]	1.00	1.64 (0.80-3.36)	2.24 (1.32-3.80)**	1.25 (0.67-2.35)	1.78 (0.93-3.41)	0.11	1.12 (0.91-1.39)	0.51
Pattern #3[‡f]	*Full*[h]	1.00	1.36 (0.80-2.31)	0.71 (0.40-1.26)	0.90 (0.50-1.61)	1.04 (0.61-1.75)	0.54	0.97 (0.81-1.18)	0.88
HEI-2015[g]	*Full*[h]	1.00	1.20 (0.73-1.99)	0.83 (0.49-1.40)	1.11 (0.66-1.86)	0.73 (0.36-1.48)	0.31	0.89 (0.72-1.11)	0.83
Cancer-Specific Mortality									
Pattern #1[†d]	*Full*[h]	1.00	0.91 (0.49-1.67)	0.72 (0.33-1.59)	0.91 (0.43-1.91)	2.03 (1.00-4.12)*	0.16	1.17 (0.86-1.60)	0.90
Pattern #2[‡e]	*Full*[h]	1.00	1.34 (0.59-3.04)	2.10 (1.10-4.01)*	0.58 (0.21-1.62)	1.95 (0.88-4.30)	0.23	1.06 (0.82-1.37)	0.91
Pattern #3[‡f]	*Full*[h]	1.00	0.57 (0.30-1.06)	0.70 (0.27-1.84)	0.86 (0.41-1.84)	1.26 (0.67-2.37)	0.26	1.14 (0.86-1.52)	0.13
HEI-2015[g]	*Full*[h]	1.00	1.00 (0.42-2.38)	0.69 (0.27-1.72)	0.93 (0.43-2.02)	0.62 (0.26-1.50)	0.26	0.89 (0.69-1.15)	0.60

** $p < 0.01$; * $p < 0.05$

[a] Test for trend across the quintiles of the dietary exposure. See Equation 2 in the main text.

[b] Hazard ratio for a standard deviation increase in the dietary exposure. See Equation 3 in the main text.

[c] Likelihood ratio test p-value for a natural cubic spline model (Equation 4 in the main text) compared to specifying the model with the scaled dietary exposure (Equation 3).

[d] The High Fat & Sugar, Low Vegetable Pattern; [e] The High Alcohol & Added Sugar Pattern; [f] The Meat & Potatoes Pattern; [g] Healthy Eating Index 2015

[h] Adjusts for age, sex, race and ethnicity, BMI, household size, family income-to-poverty ratio, education status, health insurance status, receipt of SNAP benefits, food insecurity status, alcohol intake, smoking status, total caloric intake, weekly MET minutes, and the Charlson Comorbidity Index score.

[†] Dietary pattern obtained using penalized logistic regression; [‡] Dietary pattern obtained using principal components analysis (PCA).

Subjects were weighted, and the analysis was performed according to NCHS guidelines.

This survival analysis was performed on a subset with complete data for the NHANES ADL score (n = 905) from the testing subsample described in the main text (n = 1745). All dietary patterns extraction procedures were performed on the training subsample described in the main text (n = 748).

**Supplementary Table 3**. Adjusted hazard ratios (HRs) and 95% confidence intervals from the *Full*[h] model for the risks of all-cause and cause-specific mortalities, in relation to the dietary patterns, in the sample of cancer survivors from the testing subsample who received their primary cancer diagnosis ≤ 4 years prior to the survey date (n = 535).								
**Dietary Pattern**	**Q1**	**Q2**	**Q3**	**Q4**	**Q5**	p^{a}_{trend}	$HR^{b}_{continuous}$	$p^{c}_{non\text{-}linear}$
All-Cause Mortality								
Pattern #1[†d]	1.00	1.19 (0.63-2.24)	1.68 (0.85-3.32)	1.69 (0.83-3.45)	1.29 (0.65-2.56)	0.17	1.28 (1.01-1.63)*	0.44
Pattern #2[‡e]	1.00	1.21 (0.76-1.92)	1.19 (0.70-2.03)	1.06 (0.66-1.70)	1.05 (0.61-1.79)	0.96	1.01 (0.84-1.22)	0.81
Pattern #3[‡f]	1.00	1.32 (0.77-2.28)	1.32 (0.78-2.21)	1.33 (0.77-2.31)	1.86 (1.07-3.23)*	0.05*	1.27 (1.03-1.56)*	0.84
HEI-2015[g]	1.00	1.18 (0.61-2.27)	1.00 (0.52-1.92)	1.06 (0.47-2.37)	0.80 (0.42-1.52)	0.34	0.87 (0.71-1.08)	0.72
Cancer-Specific Mortality								
Pattern #1[†d]	1.00	0.94 (0.26-3.44)	2.40 (0.70-8.23)	2.23 (0.53-9.48)	1.63 (0.64-4.13)	0.13	1.25 (0.91-1.72)	0.67
Pattern #2[‡e]	1.00	1.36 (0.64-2.88)	1.24 (0.52-2.97)	0.67 (0.30-1.49)	1.38 (0.60-3.20)	0.86	1.04 (0.78-1.38)	0.72
Pattern #3[‡f]	1.00	1.77 (0.63-4.95)	1.26 (0.52-3.03)	1.19 (0.41-3.52)	0.98 (0.26-3.73)	0.83	1.06 (0.73-1.56)	0.95
HEI-2015[g]	1.00	0.72 (0.21-2.38)	0.90 (0.33-2.47)	0.98 (0.38-2.54)	0.66 (0.29-1.46)	0.48	0.87 (0.66-1.13)	0.51

** $p < 0.01$; * $p < 0.05$

[a] Test for trend across the quintiles of the dietary exposure. See Equation 2 in the main text.

[b] Hazard ratio for a standard deviation increase in the dietary exposure. See Equation 3 in the main text.

[c] Likelihood ratio test p-value for a natural cubic spline model (Equation 4 in the main text) compared to specifying the model with the scaled dietary exposure (Equation 3).

[d] The High Fat & Sugar, Low Vegetable Pattern; [e] The High Alcohol & Added Sugar Pattern; [f] The Meat & Potatoes Pattern; [g] Healthy Eating Index 2015

[h] Adjusts for age, sex, race and ethnicity, BMI, household size, family income-to-poverty ratio, education status, health insurance status, receipt of SNAP benefits, food insecurity status, alcohol intake, smoking status, total caloric intake, weekly MET minutes, and the Charlson Comorbidity Index score.

[†] Dietary pattern obtained using penalized logistic regression; [‡] Dietary pattern obtained using principal components analysis (PCA).

Subjects were weighted, and the analysis was performed according to NCHS guidelines.

This survival analysis was performed on a subset of cancer survivors ≤ 4 years removed from their primary diagnosis (n = 535) from the testing subsample described in the main text (n = 1745). All dietary patterns extraction procedures were performed on the training subsample described in the main text (n = 748).

Supplementary Table 4. Adjusted hazard ratios (HRs) and 95% confidence intervals from the *Full*[h] model for the risks of all-cause and cause-specific mortalities, in relation to the dietary patterns, in propensity score-matched samples from the testing subsample.

**Dietary Pattern**	***n***	**Q1**	**Q2**	**Q3**	**Q4**	**Q5**	p^{a}_{trend}	$HR^{b}_{continuous}$	$p^{c}_{non-linear}$
All-Cause Mortality									
Pattern #1[†d]	1718	1.00	0.72 (0.43-1.23)	1.04 (0.70-1.55)	1.21 (0.81-1.81)	1.52 (0.98-2.34)	0.02*	1.24 (1.08-1.43)**	0.56
Pattern #2[‡e]	1710	1.00	1.84 (1.16-2.92)**	1.87 (1.15-3.04)*	1.96 (1.17-3.30)*	1.93 (1.14-3.29)*	0.01*	1.15 (1.01-1.30)*	< 0.01**
Pattern #3[‡f]	1716	1.00	0.97 (0.67-1.41)	0.74 (0.48-1.16)	0.86 (0.54-1.36)	0.80 (0.52-1.24)	0.26	0.89 (0.76-1.04)	0.45
HEI-2015[g]	1340	1.00	0.94 (0.57-1.54)	1.22 (0.76-1.98)	0.97 (0.63-1.49)	0.77 (0.47-1.29)	0.24	0.92 (0.79-1.07)	0.53
Cancer-Specific Mortality									
Pattern #1[†d]	1718	1.00	0.82 (0.40-1.66)	0.89 (0.50-1.60)	1.18 (0.62-2.25)	2.15 (1.26-3.67)**	0.02*	1.34 (1.09-1.64)**	0.98
Pattern #2[‡e]	1710	1.00	1.16 (0.58-2.34)	1.88 (0.94-3.75)	1.59 (0.73-3.43)	2.23 (1.13-4.38)*	0.01*	1.15 (0.99-1.33)	0.08
Pattern #3[‡f]	1716	1.00	0.70 (0.41-1.20)	0.68 (0.35-1.33)	0.88 (0.52-1.47)	0.87 (0.52-1.47)	0.79	1.00 (0.79-1.26)	0.19
HEI-2015[g]	1340	1.00	0.69 (0.32-1.48)	0.95 (0.38-2.40)	1.04 (0.56-1.94)	0.60 (0.27-1.32)	0.38	0.88 (0.71-1.08)	0.57

** $p < 0.01$; * $p < 0.05$
[a] Test for trend across the quintiles of the dietary exposure. See Equation 2 in the main text.
[b] Hazard ratio for a standard deviation increase in the dietary exposure. See Equation 3 in the main text.
[c] Likelihood ratio test *p*-value for a natural cubic spline model (Equation 4 in the main text) compared to specifying the model with the scaled dietary exposure (Equation 3).
[d] The High Fat & Sugar, Low Vegetable Pattern; [e] The High Alcohol & Added Sugar Pattern; [f] The Meat & Potatoes Pattern; [g] Healthy Eating Index 2015
[h] Adjusts for age, sex, race and ethnicity, BMI, household size, family income-to-poverty ratio, education status, health insurance status, receipt of SNAP benefits, food insecurity status, alcohol intake, smoking status, total caloric intake, weekly MET minutes, and the Charlson Comorbidity Index score.
† Dietary pattern obtained using penalized logistic regression; ‡ Dietary pattern obtained using principal components analysis (PCA).
Subjects were weighted, and the analysis was performed according to NCHS guidelines.
This survival analysis was performed on a subset of cancer survivors from the testing subsample with only matched samples (see supplementary methods for more details on the propensity score matching procedure).
All dietary patterns extraction procedures were performed on the training subsample described in the main text (*n* = 748).

Supplementary Table 5. Adjusted hazard ratios (HRs) and 95% confidence intervals from the *Full*[h] model for the risks of all-cause and cause-specific mortalities, in relation to the dietary patterns, in propensity score-matched samples from the testing subsample. Only food insecure cancer survivors from the testing subsample were included in this subanalysis.

**Dietary Pattern**	***n***	**Q1**	**Q2**	**Q3**	**Q4**	**Q5**	p^{a}_{trend}	$HR^{b}_{continuous}$	$p^{c}_{non\text{-}linear}$
All-Cause Mortality									
Pattern #1[†d]	210	1.00	1.41 (0.15-12.94)	4.74 (0.64-35.03)	3.70 (0.58-23.39)	1.46 (0.18-11.71)	0.74	1.20 (0.68-2.14)	0.84
Pattern #2[‡e]	204	1.00	2.00 (0.33-12.29)	1.21 (0.23-6.33)	1.81 (0.30-10.85)	0.79 (0.15-4.05)	0.59	0.89 (0.60-1.32)	0.36
Pattern #3[‡f]	208	1.00	1.47 (0.37-5.73)	0.34 (0.08-1.46)	1.05 (0.12-8.89)	0.70 (0.13-3.76)	0.45	0.81 (0.48-1.36)	0.80
HEI-2015[g]	146	1.00	0.99 (0.27-3.62)	1.08 (0.20-5.87)	0.53 (0.12-2.41)	1.89 (0.21-16.84)	0.77	0.81 (0.38-1.74)	0.53
Cancer-Specific Mortality									
Pattern #1[†d]	210	1.00	38.54 (2.60-571.83)**	36.04 (3.49-371.76)**	5.54 (0.17-178.14)	18.08 (0.88-372.96)	0.44	1.30 (0.55-3.10)	0.50
Pattern #2[‡e]	204	1.00	0.15 (0.00-6.45)	0.00 (0.00-1.23)	0.09 (0.00-6.07)	0.01 (0.00-5.61)	0.21	0.14 (0.02-1.20)	0.85
Pattern #3[‡f]	208	1.00	0.78 (0.17-3.64)	0.17 (0.01-1.99)	0.49 (0.02-10.75)	0.00 (0.00-0.02)**	0.02*	0.34 (0.12-0.99)*	0.42
HEI-2015[g]	146	1.00	83.58 (3.47-2010.75)**	168.25 (3.57-7928.71)**	8.59 (0.56-132.38)	0.13 (0.00-7.37)	0.97	0.61 (0.16-2.37)	0.13

** $p < 0.01$; * $p < 0.05$

[a] Test for trend across the quintiles of the dietary exposure. See Equation 2 in the main text.

[b] Hazard ratio for a standard deviation increase in the dietary exposure. See Equation 3 in the main text.

[c] Likelihood ratio test *p*-value for a natural cubic spline model (Equation 4 in the main text) compared to specifying the model with the scaled dietary exposure (Equation 3).

[d] The High Fat & Sugar, Low Vegetable Pattern; [e] The High Alcohol & Added Sugar Pattern; [f] The Meat & Potatoes Pattern; [g] Healthy Eating Index 2015

[h] Adjusts for age, sex, race and ethnicity, BMI, household size, family income-to-poverty ratio, education status, health insurance status, receipt of SNAP benefits, food insecurity status, alcohol intake, smoking status, total caloric intake, weekly MET minutes, and the Charlson Comorbidity Index score.

[†] Dietary pattern obtained using penalized logistic regression; [‡] Dietary pattern obtained using principal components analysis (PCA).

Subjects were weighted, and the analysis was performed according to NCHS guidelines.

This survival analysis was performed on a subset of food insecure cancer survivors from the testing subsample with only matched samples (see supplementary methods for more details on the propensity score matching procedure).

All dietary patterns extraction procedures were performed on the training subsample described in the main text (n = 748).
